# Onboard Robust Visual Tracking for UAVs Using a Reliable Global-Local Object Model

**DOI:** 10.3390/s16091406

**Published:** 2016-08-31

**Authors:** Changhong Fu, Ran Duan, Dogan Kircali, Erdal Kayacan

**Affiliations:** 1 School of Mechanical and Aerospace Engineering, Nanyang Technological University (NTU), 50 Nanyang Avenue, Singapore 639798, Singapore; changhongfu@ntu.edu.sg (C.F.); duanran@ntu.edu.sg (R.D.); dkircali@ntu.edu.sg (D.K.); 2 ST Engineering-NTU Corporate Laboratory, Nanyang Technological University, 50 Nanyang Avenue, Singapore 639798, Singapore

**Keywords:** unmanned aerial vehicle, visual object tracking, reliable global-local model, local geometric filter, local outlier factor, robust real-time performance

## Abstract

In this paper, we present a novel onboard robust visual algorithm for long-term arbitrary 2D and 3D object tracking using a reliable global-local object model for unmanned aerial vehicle (UAV) applications, e.g., autonomous tracking and chasing a moving target. The first main approach in this novel algorithm is the use of a global matching and local tracking approach. In other words, the algorithm initially finds feature correspondences in a way that an improved binary descriptor is developed for global feature matching and an iterative Lucas–Kanade optical flow algorithm is employed for local feature tracking. The second main module is the use of an efficient local geometric filter (LGF), which handles outlier feature correspondences based on a new forward-backward pairwise dissimilarity measure, thereby maintaining pairwise geometric consistency. In the proposed LGF module, a hierarchical agglomerative clustering, i.e., bottom-up aggregation, is applied using an effective single-link method. The third proposed module is a heuristic local outlier factor (to the best of our knowledge, it is utilized for the first time to deal with outlier features in a visual tracking application), which further maximizes the representation of the target object in which we formulate outlier feature detection as a binary classification problem with the output features of the LGF module. Extensive UAV flight experiments show that the proposed visual tracker achieves real-time frame rates of more than thirty-five frames per second on an i7 processor with 640 × 512 image resolution and outperforms the most popular state-of-the-art trackers favorably in terms of robustness, efficiency and accuracy.

## 1. Introduction

Visual tracking, as one of the most active vision-based research topics, can assist unmanned aerial vehicles (UAVs) to achieve autonomous flights in different types of civilian applications, e.g., infrastructure inspection [1], person following [2] and aircraft avoidance [3]. Although numerous visual tracking algorithms have recently been proposed in the computer vision community [4,5,6,7,8,9], onboard visual tracking of freewill arbitrary 2D or 3D objects for UAVs remains as a challenging task due to object appearance changes caused by a number of situations, inter alia, shape deformation, occlusion, various surrounding illumination, in-plane or out-of-plane rotation, large pose variation, onboard mechanical vibration, wind disturbance and aggressive UAV flight.

To track an arbitrary object with a UAV, the following four basic requirements should be considered to implement an onboard visual tracking algorithm: (1) real-time: the tracking algorithm must process onboard captured live image frames at high speed; (2) accuracy: the tracking algorithm must track the object accurately even with the existence of the aforementioned challenging factors; (3) adaptation: the tracking algorithm must adapt real object appearance online; (4) recovery: the tracking algorithm must be capable of re-detecting the object when the target object becomes visible in the field of view (FOV) of the camera again after the object is lost.

In this paper, the following three main modules are proposed to have a reliable visual object model under the aforementioned challenging situations and to achieve those basic requirements in different UAV tracking applications:

- A global matching and local tracking (GMLT) approach has been developed to initially find the FAST [10] feature correspondences, i.e., an improved version of the BRIEF descriptor [11] is developed for global feature matching, and an iterative Lucas–Kanade optical flow algorithm [12] is employed for local feature tracking between two onboard captured consecutive image frames based on a forward-backward consistency evaluation method [13].
- An efficient local geometric filter (LGF) module has been designed for the proposed visual feature-based tracker to detect outliers from global and local feature correspondences, i.e., a novel forward-backward pairwise dissimilarity measure has been developed and utilized in a hierarchical agglomerative clustering (HAC) approach [14] to exclude outliers using an effective single-link approach.
- A heuristic local outlier factor (LOF) [15] module has been implemented for the first time to further remove outliers, thereby representing the target object in vision-based UAV tracking applications reliably. The LOF module can efficiently solve the chaining phenomenon generated from the LGF module, i.e., a chain of features is stretched out with long distances regardless of the overall shape of the object, and the matching confusion problem caused by the multiple moving parts of objects.

Extensive UAV flight experiments show that the proposed visual tracker achieves real-time frame rates of more than thirty-five frames per second on an i7 processor with 640 × 512 image resolution and outperforms the most popular state-of-the-art trackers favorably in terms of robustness, efficiency and accuracy.

The outline of the paper is organized as follows: Section 2 presents the recent works related to the visual object tracking for UAVs. Section 3 introduces the proposed novel visual object tracking algorithm. The performance evaluations in various UAV flight tests and its comparisons with the most popular state-of-the-art visual trackers are discussed in Section 4. Finally, the concluding remarks are given in Section 5.

## 2. Related Works

### 2.1. Color Information-Based Method

Color information on the image frame has played an important role in visual tracking applications. A color-based visual tracker is proposed in [16] for UAVs to autonomously chase a moving red car. A visual tracking approach based on the color information is developed in [17] for UAVs to follow a red 3D flying object. Similarly, a color-based detection approach is employed in [18] for UAVs to track a red hemispherical airbag and to achieve autonomous landing. Although all of these color-based visual tracking approaches are very efficient and various kinds of color spaces can be adopted, this type of visual tracker is very sensitive to illumination changes and noise on the image, and it is preferably applicable for target tracking with a monotone and distinctive color.

### 2.2. Direct or Feature-Based Approach

A static or moving object tracked by a UAV has often been represented by a rectangle bounding box. Template matching (TM) has usually been applied to visual object tracking tasks for UAVs. It searches a region of interest (ROI) in the current image frame that is similar to the template defined in the first image frame. The TM approach for UAVs can be categorized into two groups: the direct method [19] and featured-based approach [20,21,22,23]. The direct method uses the intensity information of pixels directly to represent the tracked object and to track the target object, whereas the feature-based approach adopts a visual feature, e.g., Harris corner [24], SIFT [25], SURF [26] or ORB [27] feature, to track the target object. However, these existing visual trackers are not robust to the aforementioned challenging situations since the object appearance is only defined or fixed in the first image frame, i.e., they cannot update the object appearance during the UAV operation. What is more, these trackers are not suitable for tracking 3D or deformable objects.

### 2.3. Machine Learning-Based Method

Machine learning methods have also been applied to vision-based UAV tracking applications. In general, these approaches can be divided into two categories based on the learning methods: offline and online learning approaches.

#### 2.3.1. Offline Machine Learning-Based Approach

An offline-trained face detector is utilized in [28] to detect the face of a poacher from a UAV in order to protect the wildlife in Africa. An offline-learned visual algorithm is applied in [29] to detect a 2D planar target object for a UAV to realize autonomous landing. A supervised learning approach is presented in [30] to detect and classify different types of electric towers for UAVs. However, a large number of image training datasets, i.e., positive and negative image patches with all aforementioned challenging conditions, should be cropped and collected to train these trackers, thereby guaranteeing their high detection accuracies. Moreover, labeling the image training datasets requires much experience, and it is a tedious, costly and time-consuming task. In addition, an offline-trained visual tracker is only capable of tracking specifically-trained target objects instead of freewill arbitrary objects.

#### 2.3.2. Online Machine Learning-Based Method

Recently, online learning visual trackers have been developed as the most promising tracking approaches to track an arbitrary 2D or 3D object. Online learning visual tracking algorithms are generally divided into two categories: generative and discriminative methods.

Generative approaches learn only a 2D or 3D tracking object online without considering the background information around the tracking object and then apply the online-learned model for searching the ROI on the current image frame with minimum reconstruction error. Visual tracking algorithms based on incremental subspace learning [31] and hierarchical methods are developed in [28,32] to track a 2D or 3D object for UAVs. Although these works obtained promising tracking results, a number of object samples from consecutive image frames should be collected, and they have assumed that the appearance of the target object does not change significantly during the image collection period.

Discriminative methods treat the tracking problem as a binary classification task to separate the object from its background using an online updating classifier with positive and negative (i.e., background) information. A tracking-learning-detection (TLD) [8] approach is utilized in [33] for a UAV to track different objects. A real-time adaptive visual tracking algorithm, which is based on a vision-based compressive tracking approach [6], is developed in [1] for UAVs to track arbitrary 2D or 3D objects. A structured output tracking with kernels (STRUCK) algorithm is adopted in [2] for a UAV to follow a walking person. However, updating on consecutive image frames is prone to include noises, and the drift problem is likely to occur, thereby resulting in tracking failure. Although an online multiple-instance learning (MIL) [4] approach is developed in [3] to improve tracking performance for UAVs, the update step may still not effectively eliminate noises. Additionally, most of the discriminative methods cannot estimate the scale changes of the target object.

## 3. Proposed Method

The proposed method in this paper mainly includes three modules: (1) global matching and local tracking (GMLT); (2) local geometric filter (LGF); and (3) local outlier factor (LOF).

### 3.1. Global Matching and Local Tracking Module

**Figure 1:**
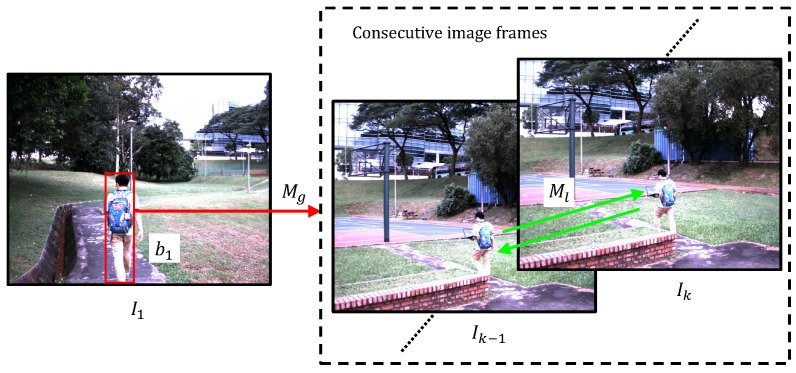
Illustration of the global matching and local tracking (GMLT) module. The bounding box $b_{1}$ is shown with the red rectangle. The FAST features detected in the $b_{1}$ compose the global object model $M_{g}$, which is employed to globally find the feature correspondences on onboard captured consecutive image frames with the improved BRIEF descriptor. The green arrow is the Lucas–Kanade optical flow algorithm-based tracking between the (*k*-1) -th frame and the *k*-th frame. $M_{l}$ is the local object model, which is updated frame-by-frame.

Let $b_{1}$ be a bounding box around an online selected 2D or 3D target object, e.g., a moving person on the first image frame $I_{1}$ shown in Figure 1. The FAST features on each RGB image frame are detected using a bucketing approach [34], i.e., the captured image frame is separated into pre-defined non-overlapped rectangle regions, i.e., buckets. The bucketing approach keeps the FAST features evenly distributed in the image frame and guarantees the real-time visual tracking performance. A group of the FAST features detected in $b_{1}$ is denoted as $\left\{x_{1}^{1},x_{1}^{2},\ldots ,x_{1}^{n}\right\}$, where $x_{1}^{i}\in R^{2}$, i=1,2,…,n; they compose the global model $M_{g}$ of the target object. The model $M_{g}$ is utilized to globally match the candidate FAST features detected on each image frame with the improved version of the BRIEF descriptor. It is to be noted that the global matching is able to achieve the re-detection of the object when the target object becomes visible in the camera FOV again after the object is lost. For the *i*-th FAST feature on the *k*-th image frame $x_{k}^{i}$, its improved BRIEF descriptor, i.e., $B\left(x_{k}^{i}\right)=\left\{B_{1}\left(x_{k}^{i}\right),B_{2}\left(x_{k}^{i}\right),\ldots ,B_{N_{b}}\left(x_{k}^{i}\right)\right\}$, is defined as follows:

(1) $$B_{j}\left(x_{k}^{i}\right)=\left\{\begin{array}{cc} 1 & \text{if}\;I_{k}(x_{k}^{i}\;+\;p_{j})\;<\;I_{k}(x_{k}^{i}\;+\;q_{j})\\ 0 & \text{otherwise} \end{array}\right.,\forall j\in \left[1,\ldots ,N_{b}\right]$$

where $B_{j}\left(x_{k}^{i}\right)$ is the *j*-th bit of the binary vector in $B(x_{k}^{i})$, $I_{k}\left(*\right)$ is the intensity of the pixel on the *k*-th image frame and ($p_{j},q_{j}$) is sampled in a local neighbor region $S_{r}\times S_{r}$ based on the location of the *i*-th FAST feature. $p_{j}=N\left(0,{\left(\frac{1}{5}S_{r}\right)}^{2}\right)$ and $q_{j}=N\left(p_{j},{\left(\frac{2}{25}S_{r}\right)}^{2}\right)$. If the intensity of the pixel on the location $x_{k}^{i}$ + $p_{j}$ is smaller than the one on the location $x_{k}^{i}$ + $q_{j}$, then the $B_{j}\left(x_{k}^{i}\right)$ is one, otherwise, it is zero. The parameter $N_{b}$ is the length of the binary vector $B(x_{k}^{i})$, i.e., the number of comparisons to perform. It is to be noted that the distance *d* of two binary vectors is computed by counting the number of different bits between these two vectors, i.e., the Hamming distance, which has less computation cost compared to the Euclidean distance. For searching the FAST feature correspondences on the *k*-th (k≥2) image frame using the model $M_{g}$, the FAST feature that has the lowest Hamming distance $d_{1}$ is the best feature candidate; the FAST feature that has the second-lowest Hamming distance $d_{2}$ is the second-best feature candidate; when the ratio between the best and second-best match $d_{1}/d_{2}$ is less than a threshold *ρ*, the best FAST feature candidate is accepted as a matched FAST feature.

An iterative Lucas–Kanade optical flow algorithm with a three-level pyramid has been utilized to track each FAST feature between the (*k*-1)-th image frame and the *k*-th image frame based on the forward-backward consistency evaluation approach within a $S_{h}\times S_{h}$ local search window. These tracked FAST features constitute the local model $M_{l}$ of the target object. It is to be noted that in the local tracking stage, the model $M_{l}$ is updated frame-by-frame, i.e., the model $M_{l}$ is adaptive.

Let $F_{1}$ be the matched and tracked FAST features on the *k*-th image frame, denoted as $F_{1}=\left\{x_{k}^{1},x_{k}^{2},\ldots ,x_{k}^{m}\right\}$, where $x_{k}^{i}\in R^{2}$, i=1,2,…,m. In this work, the scale $s_{k}$ of the target object is estimated based on [13], i.e., for each pair of FAST features, a ratio between the FAST feature distance on the current image frame and the corresponding FAST feature distance on the first image frame is calculated:

(2) $$s_{k}^{ij}=\frac{\left|\right|x_{k}^{i}-x_{k}^{j}\left|\right|}{\left|\right|x_{1}^{i}-x_{1}^{j}\left|\right|},i\neq j$$

and then, the median of $\left\{s_{k}^{ij}\right\}$ is the estimated scale $s_{k}$ of the target object, since it is more robust with respect to outliers.

**Figure 2:**
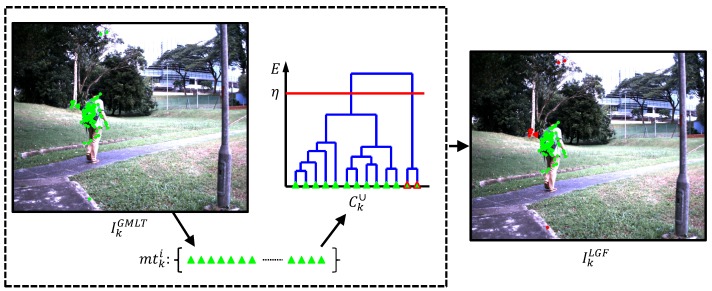
Illustration of the local geometric filter (LGF) module. The green points on the $I_{k}^{GMLT}$ are the matched and tracked FAST features from the GMLT module, i.e., $F_{1}$. The green triangles are the FAST feature correspondences, i.e., $C_{k}^{\cup }$. The LGF module is utilized to filter outlier correspondences, as the green triangles with red edges shown in the dendrogram. The red points on the $I_{k}^{LGF}$ are the outliers filtered by the LGF module.

In our extensive visual tracking tests, we find that the $F_{1}$ includes certain outlier FAST features, as one example is shown in the left image in Figure 2, i.e., $I_{k}^{GMLT}$. Let a matching with the model $M_{g}$ be a global FAST feature correspondence, a tracking with model $M_{l}$ be a local FAST feature correspondence and the combination of the global matching and local tracking correspondences be $C_{k}^{\cup }$; ${mt}_{k}^{i}$ is the *i*-th FAST feature correspondence in $C_{k}^{\cup }$. The next subsection introduces the second main module to detect these outliers.

### 3.2. Local Geometric Filter Module

**Figure 3:**
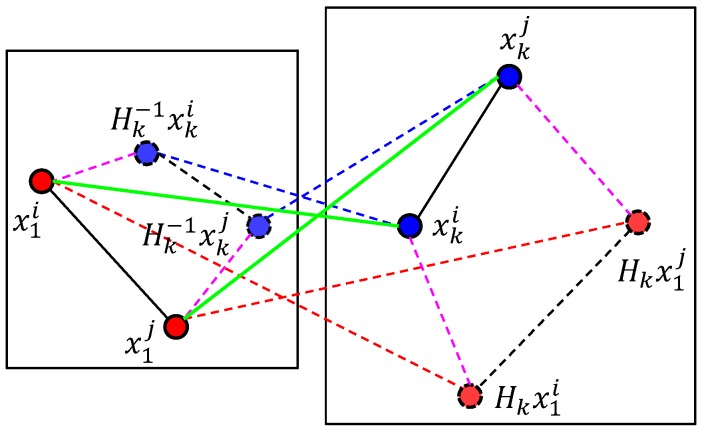
Forward-backward pairwise dissimilarity measure $E_{\text{LGF}}$ between correspondence $mt_{k}^{i}$ and $mt_{k}^{j}$ (green solid lines). The dashed red and blue points are transformed by the homography $H_{k}$ and the inversion of homography $H_{k}^{-1}$.

The second main module in the proposed method is a novel efficient local geometric filter (LGF), which utilizes a new forward-backward pairwise dissimilarity measure $E_{\text{LGF}}$ between correspondences $mt_{k}^{i}$ and $mt_{k}^{j}$ based on pairwise geometric consistency, as illustrated in Figure 3.

The $E_{\text{LGF}}$ for every pair of correspondences is defined as below:

(3) $$E_{\text{LGF}}\left(mt_{k}^{i},mt_{k}^{j}\right)=\frac{1}{2}\left[E_{\text{LGF}}\left(mt_{k}^{i},mt_{k}^{j}|H_{k}\right)+E_{\text{LGF}}\left(mt_{k}^{i},mt_{k}^{j}|H_{k}^{-1}\right)\right],i\neq j$$

where:

$$E_{\text{LGF}}\left(mt_{k}^{i},mt_{k}^{j}|H_{k}\right)=\left\|\left(x_{k}^{i}-x_{k}^{j}\right)-H_{k}\left(x_{1}^{i}-x_{1}^{j}\right)\right\|,i\neq j$$

$$E_{\text{LGF}}\left(mt_{k}^{i},mt_{k}^{j}|H_{k}^{-1}\right)=\left\|\left(x_{1}^{i}-x_{1}^{j}\right)-H_{k}^{-1}\left(x_{k}^{i}-x_{k}^{j}\right)\right\|,i\neq j$$

‖∗‖ is the Euclidean distance; $H_{k}$ is a homography transformation [35] estimated by the $C_{k}^{\cup }$; and $H_{k}^{-1}$ is the inversion of this homography transformation.

To reduce the ambiguity correspondences and filter the erroneous correspondences, a hierarchical agglomerative clustering (HAC) approach [14] is utilized to separate outlier correspondences from inliers based on an effective single-link approach with the forward-backward pairwise dissimilarity measure $E_{\text{LGF}}$. Let $S(G,G^{\prime })$ be a cluster dissimilarity for all pairs of clusters, $G,G^{\prime }\subset C_{k}^{\cup }$; the single-link HAC algorithm is defined:

(4) $$S\left(G,G^{\prime }\right)=\min_{mt_{k}^{i}\in G,mt_{k}^{j}\in G^{\prime }}E_{\text{LGF}}\left(mt_{k}^{i},mt_{k}^{j}\right)$$

It defines the cluster dissimilarity *S* as the minimum among all of the forward-backward pairwise dissimilarities between the two correspondences of the two clusters. A dendrogram generated from the single-link HAC approach is shown in the middle of Figure 2; the $C_{k}^{\cup }$ is divided into some subgroups based on a cut-off threshold *η*, and the biggest subgroup is considered as the correspondences for the target object. The green points shown on the right side of Figure 2, i.e., $I_{k}^{LGF}$, are the FAST features output from the LGF module, denoted as $F_{2}=\left\{{\bar{x}}_{k}^{1},{\bar{x}}_{k}^{2},\ldots ,{\bar{x}}_{k}^{w}\right\}$, where ${\bar{x}}_{k}^{i}\in R^{2}$, i=1,2,…,w, while the red points are the outliers.

The bottom-up aggregation in the single link-based clustering method is strictly local. The single-link HAC approach is easy to generate the chaining phenomenon, i.e., a chain of correspondences is stretched out for long distances without considering the real shape of the target object, especially in cluttered environments, leading to inefficient exclusion of the outliers. Additionally, multiple parts of objects, e.g., moving hands, are prone to confuse the FAST feature matching in the next new image frame. In this work, the local outlier factor (LOF) is developed to handle the chaining and confusion problems efficiently. The following subsection introduces the third main module.

### 3.3. Local Outlier Factor Module

The third main module of this work is a heuristic local outlier factor (LOF) [15], which is developed for the first time in a visual tracking application to further remove outliers, thereby maximizing target object representation and solving the matching confusion problem. The LOF is based on local density, i.e., the outlier is considered when its surrounding space contains relatively few FAST features.

**Figure 4:**
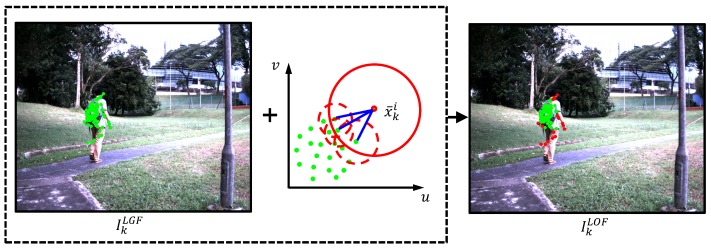
Illustration of local outlier factor (LOF) module. The green points on the $I_{k}^{LGF}$ are the FAST features from the LGF module, i.e., $F_{2}$. The LOF module is developed to further remove the outliers, as the red points shown on the $I_{k}^{LOF}$. The green points on the $I_{k}^{LOF}$ are final reliable output FAST features in our visual tracking application.

As shown in Figure 4, the local density of the FAST feature ${\bar{x}}_{k}^{i}$ is compared to the densities of its neighborhood FAST features. In this case, if the FAST feature ${\bar{x}}_{k}^{i}$ has much lower density than its neighbors, then it is an outlier.

In this work, we formulate outlier feature detection as a binary classification problem. The binary classifier is defined as follows:

(5) $$f\left({\bar{x}}_{k}^{i}\right)=\left\{\begin{array}{cc} \text{target object}, & E_{\text{LOF}}\left({\bar{x}}_{k}^{i}\right)\leq \mu \\ \text{outlier}, & E_{\text{LOF}}\left({\bar{x}}_{k}^{i}\right)>\mu \end{array}\right.$$

where $E_{\text{LOF}}\left({\bar{x}}_{k}^{i}\right)$ is a density dissimilarity measure of FAST feature ${\bar{x}}_{k}^{i}$, ${\bar{x}}_{k}^{i}\in F_{2}$, and *μ* is a cut-off threshold to classify that a FAST feature belongs to the target object or an outlier. If the value of the $E_{\text{LOF}}\left({\bar{x}}_{k}^{i}\right)$ is larger than *μ*, then ${\bar{x}}_{k}^{i}$ is the outlier, otherwise, ${\bar{x}}_{k}^{i}$ belongs to the target object.

The LOF module includes three steps to calculate the $E_{\text{LOF}}\left({\bar{x}}_{k}^{i}\right)$:

- Construction of the nearest neighbors: the nearest neighbors of the FAST feature ${\bar{x}}_{k}^{i}$ are defined as follows:
  (6) $$\mathrm{NN}\left({\bar{x}}_{k}^{i}\right)=\left\{{\bar{x}}_{k}^{j}\in F_{2}\backslash \left\{{\bar{x}}_{k}^{i}\right\}|D\left({\bar{x}}_{k}^{i},{\bar{x}}_{k}^{j}\right)\leq R_{t}\left({\bar{x}}_{k}^{i}\right)\right\}$$
  where $D({\bar{x}}_{k}^{i},{\bar{x}}_{k}^{j})$ is the Euclidean distance between the FAST features ${\bar{x}}_{k}^{i}$ and ${\bar{x}}_{k}^{j}$. $R_{t}\left({\bar{x}}_{k}^{i}\right)$ is the Euclidean distance from ${\bar{x}}_{k}^{i}$ to the *t*-th nearest FAST feature neighbor.
- Estimation of neighborhood density: the neighborhood density *δ* of the FAST feature ${\bar{x}}_{k}^{i}$ is defined as:
  (7) $$\delta \left({\bar{x}}_{k}^{i}\right)=\frac{\left|\mathrm{NN}({\bar{x}}_{k}^{i})\right|}{\sum_{{\bar{x}}_{k}^{j}\in \mathrm{NN}\left({\bar{x}}_{k}^{i}\right)}\text{max}\left\{R_{t}\left({\bar{x}}_{k}^{j}\right),\;D\left({\bar{x}}_{k}^{i},{\bar{x}}_{k}^{j}\right)\right\}}$$
  where $\left|\mathrm{NN}({\bar{x}}_{k}^{i})\right|$ is the nearest neighbor number of ${\bar{x}}_{k}^{i}$.
- Comparison of neighborhood densities: the comparison of neighborhood densities results in the density dissimilarity measure $E_{\text{LOF}}\left({\bar{x}}_{k}^{i}\right)$, which is defined below:
  (8) $$E_{\text{LOF}}\left({\bar{x}}_{k}^{i}\right)=\frac{\sum_{{\bar{x}}_{k}^{j}\in \mathrm{NN}\left({\bar{x}}_{k}^{i}\right)}\frac{\delta ({\bar{x}}_{k}^{j})}{\delta ({\bar{x}}_{k}^{i})}}{\left|\mathrm{NN}({\bar{x}}_{k}^{i})\right|}$$

Figure 4 shows the illustration of the LOF module; the green points on the $I_{k}^{LOF}$ are final reliable output FAST features for our visual tracking application, denoted as $F_{3}=\left\{{\hat{x}}_{k}^{1},{\hat{x}}_{k}^{2},\ldots ,{\hat{x}}_{k}^{o}\right\}$, where ${\hat{x}}_{k}^{i}\in R^{2}$, i=1,2,…,o. The FAST features in $F_{3}$ and their corresponding features in $b_{1}$ compose final FAST feature correspondences ${\hat{C}}_{k}^{\cup }$. Then, the center $c_{k}$ of the target object is calculated as follows:

(9) $$c_{k}=\frac{\sum_{mt_{k}^{i}\in {\hat{C}}_{k}^{\cup }}\left({\hat{x}}_{k}^{i}-Hx_{1}^{i}\right)}{\left|{\hat{C}}_{k}^{\cup }\right|}$$

## 4. Real Flight Tests and Comparisons

**Figure 5:**
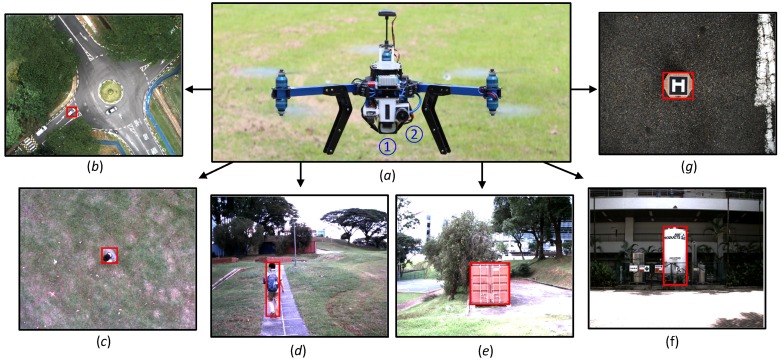
Robust real-time accurate long-term visual object tracking onboard our Y6 coaxial tricopter UAV. No. 1 and 2 in (**a**) show downward- and forward-looking monocular RGB cameras. Some 2D or 3D objects with their tracking results are shown in (**b**∓**g**).

In the UAV flight experiments, a Y6 coaxial tricopter UAV equipped with a Pixhawk autopilot from 3D Robotics is employed; the onboard computer is an Intel NUC Kit NUC5i7RYH Mini PC, which has a Core i7-5557U processor with dual-core, 16 GB RAM and a 250-GB SATA SSD drive. Both forward- and downward-looking cameras are USB 3.0 RGB cameras from Point Grey, i.e., Flea3 FL3-U3-13E4C-C, which capture the image frames with a resolution of 640 × 512 at 30 Hz. The whole UAV system is shown in Figure 5.

To practically test and evaluate the robustness, efficiency and accuracy of the proposed onboard visual tracker, we have developed our visual tracker in C++ and conducted more than fifty UAV flights in various types of environments of Nanyang Technological University, including challenging situations. As shown in Figure 5, target objects include a moving car (b), walking people (c and d), a container (e), a gas tank (f) and a moving unmanned ground vehicle (UGV) with a landing pad (g). In this paper, six recorded image sequences are randomly selected which contain 11,646 image frames, and manually labeled for the ground truth. The challenging factors of the each image sequence are listed in Table 1.

To compare our proposed visual tracker, we have employed the most popular state-of-the-art visual trackers, e.g., MIL [4], STRUCK [5], CT [6], Frag [7], TLD [8] and KCF [9], which have adaptive capabilities for appearance changes of the target objects and have been utilized to achieve the real UAV tracking applications. For all of these state-of-the-art trackers, we have utilized the source or binary programs provided by the authors with default parameters. In our proposed visual tracker, the main parameters are defined in Table 2 below. In addition, all visual trackers are initialized with the same parameters, e.g., initial object location.

**Figure 6:**
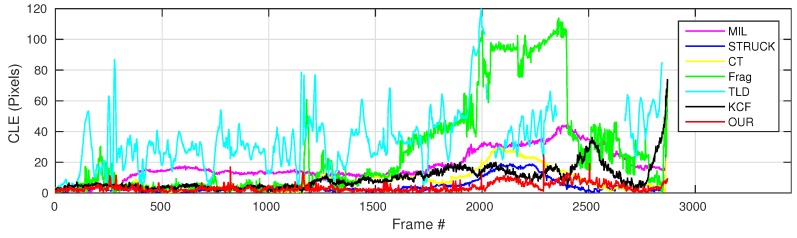
Center location error (CLE) error evolution plot of all visual trackers with the container image sequence.

**Figure 7:**
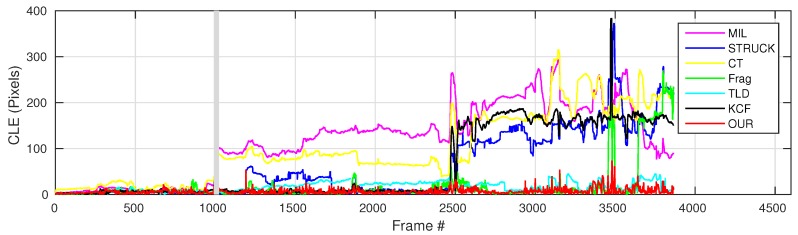
CLE evolution plot of all visual trackers with gas tank image sequence. The grey area represents that the whole target object is out of view.

**Figure 8:**
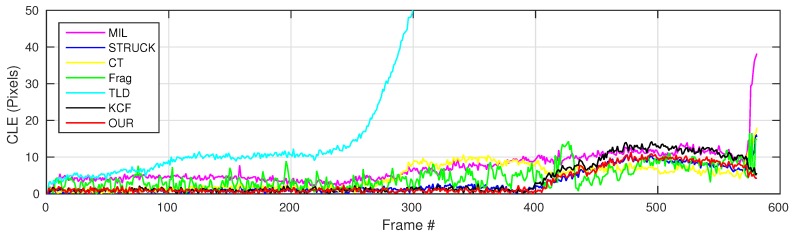
CLE error evolution plot of all trackers with the moving car image sequence.

**Figure 9:**
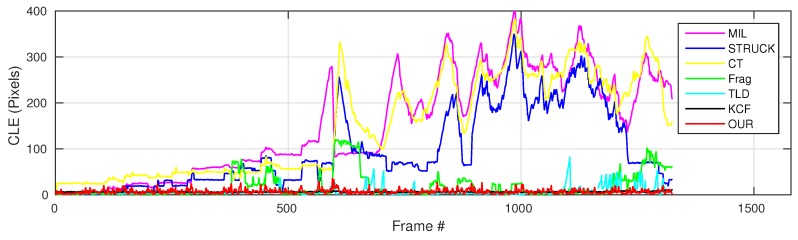
CLE error evolution plot of all trackers with the UGV_*lp*_ image sequence.

**Figure 10:**
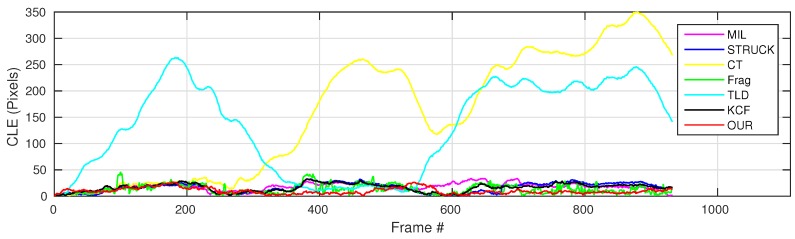
CLE error evolution plot of all trackers with the People_*bw*_ image sequence.

**Figure 11:**
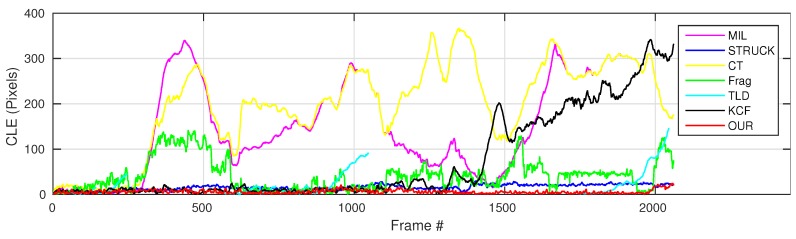
CLE error evolution plot of all trackers with the People_*fw*_ image sequence.

**Figure 12:**
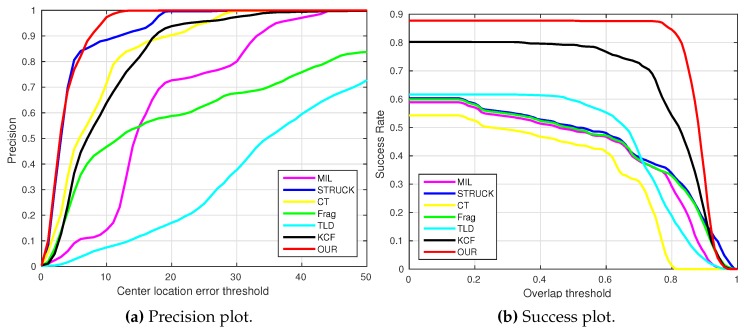
Precision and success plots of all visual trackers with the container image sequence.

**Figure 13:**
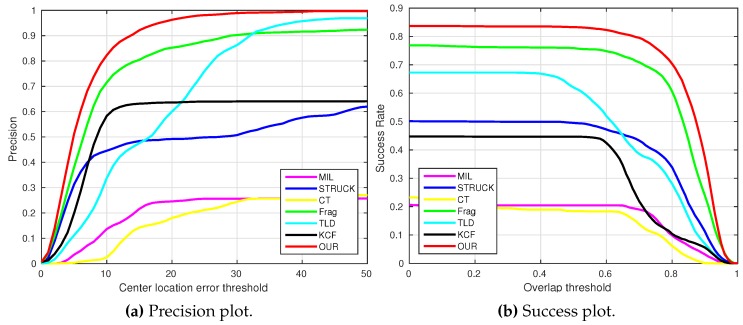
Precision and success plots of all visual trackers with the gas tank image sequence.

**Figure 14:**
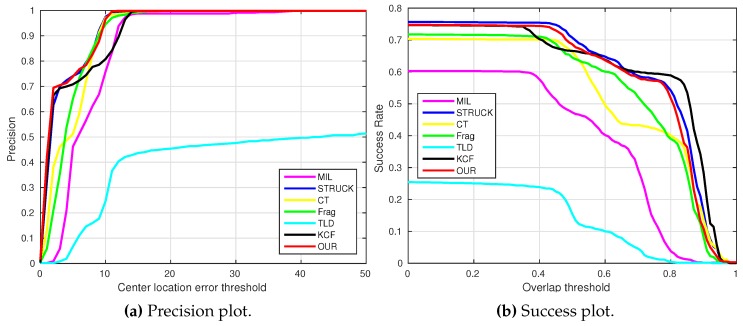
Precision and success plots of all trackers with the moving car image sequence.

**Figure 15:**
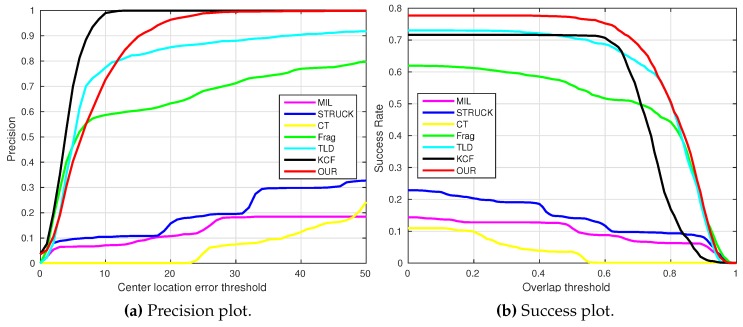
Precision and success plots of all trackers with the UGV_*lp*_ image sequence.

**Figure 16:**
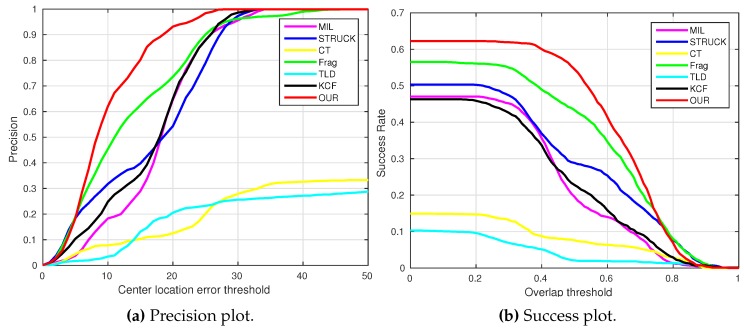
Precision and success plots of all trackers with the People_*bw*_ image sequence.

**Figure 17:**
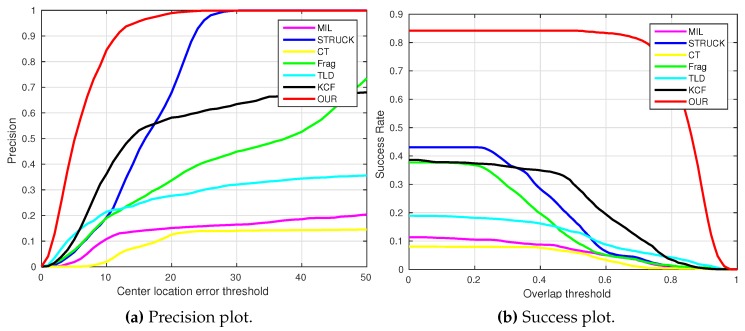
Precision and success plots of all trackers with the People_*fw*_ image sequence.

In this work, the center location error (CLE) of the tracked target object has been utilized to evaluate all visual trackers. It has been defined as the Euclidean distance between the estimated target object center and the manually-labeled ground truth center on each image frame, i.e.:

(10) $$\text{CLE}=\left\|O_{k}^{E}-O_{k}^{GT}\right\|$$

where $O_{k}^{E}$ and $O_{k}^{GT}$ are the estimated and ground truth centers of the target object. Figure 6, Figure 7, Figure 8, Figure 9, Figure 10 and Figure 11 show the CLE evolutions of all visual trackers in different image sequences. Specifically, we note that the TLD tracker easily loses the target completely for certain image frames when the target object is still in the FOV of the onboard camera; since it is able to re-detect the target object, we show the CLE error for the image sequence that the TLD tracker can track more than 96% of frames as a reference. Table 3 shows the CLE errors of all visual trackers. To visualize the tracking precisions of all visual trackers, Figure 12a, Figure 13a, Figure 14a, Figure 15a, Figure 16a and Figure 17a show the precision plots of all image sequences.

In addition, the success score (SS) has also been employed to evaluate the performances of all visual trackers in this paper, as it can evaluate the scales of the target object. The SS has been defined as below:

(11) $$\mathrm{SS}=\frac{\left|ROI_{E}\cap ROI_{GT}\right|}{\left|ROI_{E}\cup ROI_{GT}\right|}$$

where $ROI_{E}$ and $ROI_{GT}$ are the estimated and ground truth sizes of the target object. ∩ and ∪ are the intersection and union operators. |∗| represents the number of pixels in a region. If the SS is larger than *ξ* in an image frame, the tracking result is considered as a success. Table 4 shows the tracking results (*ξ* = 0.5) of all visual trackers in terms of success rate, which is defined as the ratio between the number of success frames and the total number of image frames. Moreover, we have shown the area under area (AUC) of each success plot, which is defined as the average of the success rates based on the overlap thresholds, as the results shown in Figure 12b, Figure 13b, Figure 14b, Figure 15b, Figure 16b and Figure 17b.

### 4.1. Test 1: Visual Tracking of The Container

**Figure 18:**
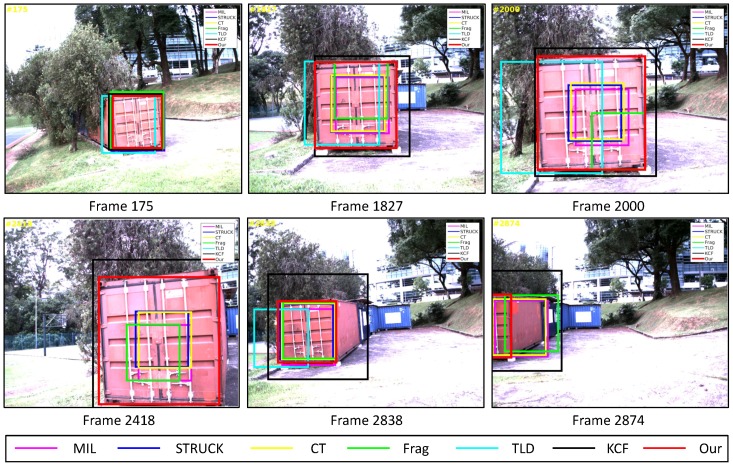
Some tracking results in the container image sequence. The total number of frames: 2874.

In this test, a static container is selected as the target object for our Y6 coaxial tricopter UAV to carry out the visual tracking application, and the onboard forward-looking camera is employed to track the locations of the target object. As the challenging factors concluded in Table 1, the container image sequence includes mechanical vibration (all image frames), scale variation (e.g., Figure 18, Frames 1827 and 2000), out-of-plane rotation (e.g., Figure 18, Frames 175 and 2418), out-of-view (e.g., Figure 18, Frame 2874) and cluttered background (all image frames).

As can be seen in Figure 6, we can find that the CLE errors of our presented visual tracker (red line) and STRUCK (blue line) are always less than 20 pixels, i.e., the precisions of these two visual trackers can achieve almost one when the CLE threshold is 20 pixels, as shown in Figure 12a. Moreover, the tracking performance of the CT tracker (yellow line) is ranking as No. 3 in this image sequence; its CLEs are changing extensively when the target object is out-of-plane; the maximum CLE error of the CT tracker is 29.8 pixels. The KCF tracker is ranking No. 4; its performance is decreasing when the flying UAV is approaching the target object. Additionally, the MIL tracker (magenta line) outperforms the TLD (cyan line) and Frag (green line) trackers, and the tracking performance of Frag is better than that of the TLD tracker; it is noticed that the TLD tracker completely loses track of the target object when some portion of the target object is out of view, i.e., some parts of the target object are not shown in the FOV of the onboard forward-looking camera, as Frame 2874 shown in Figure 18. In addition, the 2874th frame also shows that our presented visual tracker is able to locate the target object accurately even under the out-of-view situation.

Figure 12b shows the average success rates of all visual trackers. Since the MIL, STRUCK, CT and Frag trackers cannot estimate the scales of the target object, their average success rates are relatively low. Conversely, the TLD, KCF and our presented visual tracker can estimate the target object scales. However, the accuracies of the TLD and KCF trackers for estimating the center locations are lower. Therefore, the average success rates of the TLD and KCF trackers are also lower than ours.

### 4.2. Test 2: Visual Tracking of the Gas Tank

**Figure 19:**
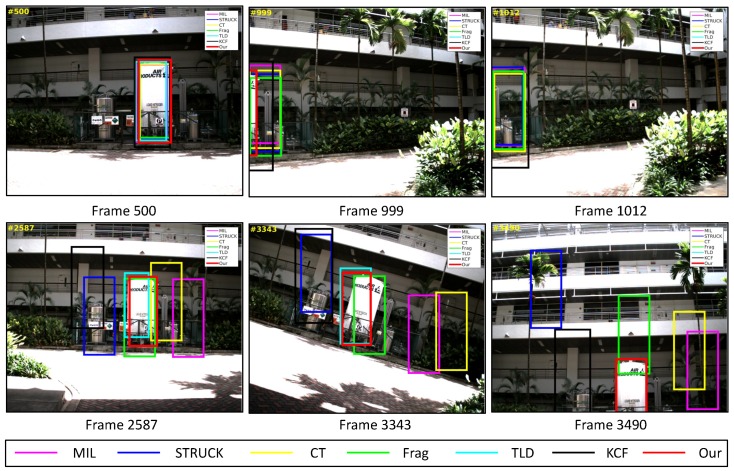
Some tracking results in the gas tank image sequence. The total number of frames: 3869.

A static gas tank is employed as the tracking object of our UAV in this test. As shown in Figure 19, this target object does not contain much texture information. Additionally, the aggressive flight (e.g., Figure 19, Frames 3343 and 3490), out-of-view (e.g., Figure 19, Frames 999, 1012 and 3490), scale variation (e.g., Figure 19, Frames 500 and 2587) and cluttered background (all of the frames) are the main challenging factors.

As shown in Figure 7, the CLE errors of the MIL and CT trackers are relatively high after the whole target object has been out-of-view (the period is shown as the gray area). In this case, the MIL and CT trackers have completely learned new appearances for their target objects, resulting in losing the target object, which they should track. Moreover, the STRUCK tracker has some drifts from Frame 1191 because of the sudden large displacement.

From Frame 2452, our UAV has carried out the first aggressive flight. The CLE errors of the STRUCK and KCF trackers started to increase due to larger displacement, since the STRUCK and KCF trackers have also adapted to the new appearances of the target object during the first aggressive flight, leading to losing their target objects until the end of the UAV tracking task.

The second aggressive flight has started from Frame 2903. Although the movements are even larger than the ones in the first aggressive flight, the Frag and our presented trackers can locate the target object well; their CLE errors are 7.8 and 7.5 pixels, respectively. The strongest aggressive flight in this test is from Frame 3318, as Frames 3343 and 3490 shown in Figure 19; its maximum flight speed has reached 3.8 m/s. Figure 13a shows the precision plot of all visual trackers. It can be seen that our presented visual tracker has achieved 90% precision when the CLE threshold is 14 pixels. In addition, Figure 13b also shows that our presented visual tracker is ranked as No.1.

### 4.3. Test 3: Visual Tracking of the Moving Car

**Figure 20:**
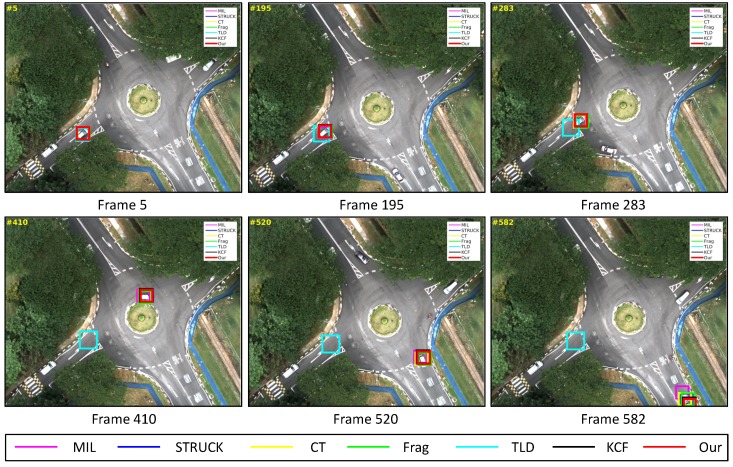
Some tracking results in the moving car image sequence. The total number of frames: 582.

In Tests 3–6, moving target objects are selected for our UAV to conduct the vision-based tracking applications. In this test, our UAV is utilized to track one moving car from an 80 m height over a traffic intersection. The main challenging factors are mechanical vibration (all image frames), in-plane rotation (e.g., Figure 20, Frame 410), out-of-view (e.g., Figure 20, Frame 582) and similar appearances of other moving cars (all image frames).

Figure 8 shows the CLE error evolutions of all visual trackers. We can find that all of the trackers can track the target object well in all image frames, except for the TLD tracker, as also shown in Figure 14a; the CT, STRUCK, Frag and our trackers have achieved 95% precision when the CLE threshold is 10.2 pixels.

From Frame 301, the TLD tracker has lost its target object completely because of its adaptation to a new target appearance, which is similar to the background information around the moving car, as shown in Figure 20, Frames 5 and 410. Additionally, the MIL and Frag trackers have generated slightly higher drifts compared to the STRUCK, CT, KCF and our tracker at the beginning of the UAV tracking application. When the moving car is conducting the in-plane rotation movement, these six trackers have started to generate larger drifts, as shown in Figure 20, Frame 520; they are not able to locate the head of the moving car. Before the moving car is out-of-view, the MIL also lost track of the moving car; it located the “HUMP” logo on the road, as shown in Figure 20, Frame 582. When some portion of the moving car is out-of-view, only KCF and our tracker can continue to locate the moving car well, achieving better tracking performances. As can be seen from Figure 14b, we can also find that the MIL, STRUCK, CT, Frag, KCF and our presented tracker have outperformed the TLD tracker.

### 4.4. Test 4: Visual Tracking of the UGV with the Landing Pad (UGV_lp_)

**Figure 21:**
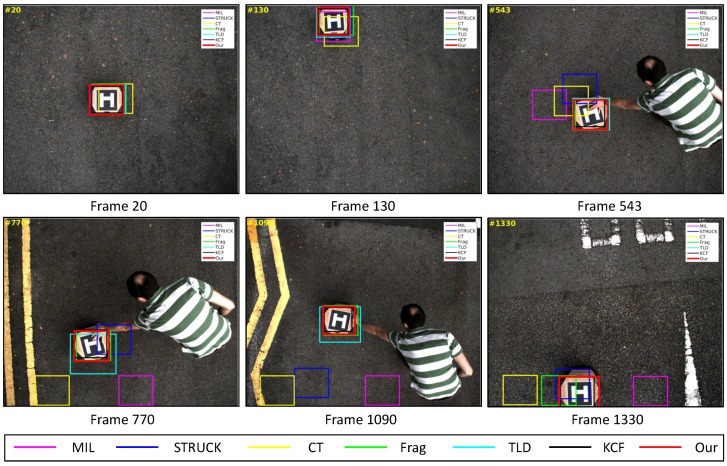
Some tracking results in the UGV_*lp*_ image sequence. The total number of frames: 1325.

In this test, a moving UGV with a landing pad is chosen. During the UAV tracking process, the direction of the UGV is manually controlled by an operator, as Frames 543, 770 and 1090 shown in Figure 21. Thus, some portion of target object is occluded by the operator’s hand.

As can be seen from Figure 9 and Figure 15, the KCF, Frag, TLD and our presented tracker have outperformed the CT, MIL and STRUCK trackers. Especially, the CT and our presented tracker have generated the drifts on Frame 3 because of the sudden roll rotation of our UAV. However, our presented tracker has tracked the target object back on Frame 4, while the CT tracker has learned a new appearance on Frame 3, i.e., the background information around the target object has been included as positive samples to train and update its model. Therefore, the CT tracker cannot locate the target object well from Frame 4. For the MIL and STRUCK trackers, their performances are similar to that of the CT tracker in a way that both of them have also adapted to the new appearances of target objects when our UAV is quickly moving forward. Although the CLE error of the KCF tracker is less than ours, the average success rate of our presented tracker is better than the one of the KCF tracker.

### 4.5. Test 5: Visual Tracking of Walking People Below (People_bw_)

**Figure 22:**
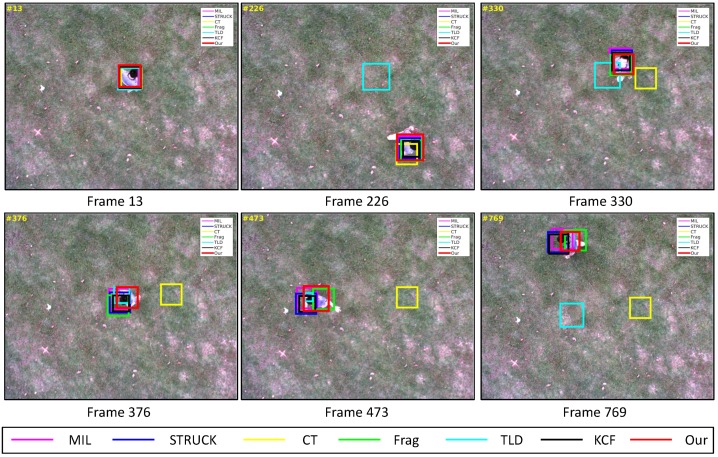
Some tracking results in the People_*bw*_ image sequence. The total number of frames: 934.

Recently, different commercial UAVs have been developed to follow a person. However, most of these UAVs still mainly depend on the GPS and IMU sensors to achieve the person following application. Therefore, a moving person is selected as the target object for our vision-based UAV tracking task in this paper. The task of the fifth test is that our UAV is utilized to locate a moving person from a high altitude using its onboard downward-looking camera. The main challenging factors include deformation and in-plane rotation, as shown in Figure 22.

As shown in Figure 10, we can find that all visual trackers except the TLD and CT trackers, can locate the moving person in all image frames. Their CLE errors are less than 46 pixels. As can be seen in Figure 16a, their precisions have achieved more than 90% when the CLE threshold is 27 pixels. For the TLD tracker, it has lost the target object from Frame 72, since it has adapted to a new appearance of the target object when the deformable target object is conducting in-plane rotation. After the target object moved back to the previous positions, the TLD tracker has learned the appearance of the target object back. Therefore, the TLD tracker can locate the target object well. From Frame 566, the TLD tracker has lost its deformable target object again until the end of the visual tracking application because of in-plane rotation. For the CT tracker, its tracking performance has also been influenced by Figure 16b; although the MIL, STRUCK, KCF and our tracker can track the target object well, their average success rates are relatively low because of in-plane rotation and deformation.

### 4.6. Test 6: Visual Tracking of Walking People in Front (People_fw_)

**Figure 23:**
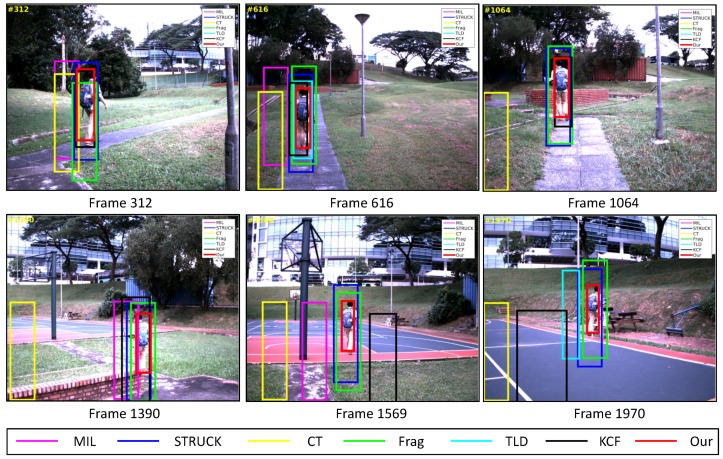
Some tracking results in the People_*fw*_ image sequence. The total number of frame: 2062.

In this test, our UAV is employed to follow a moving person using its onboard forward-looking camera. The main challenging factors include deformation, scale variation, cluttered background and out-of-plane rotation (e.g., Figure 23, Frames 312, 1390 and 1970).

As can be seen in Figure 11 and Frame 312 shown in Figure 23, the MIL and CT trackers have lost their target object because of the similar appearance in the background, e.g., a tree. Although the MIL tracker has relocated its target object from Frame 1151 and 1375, it has continued to lose the target object from Frame 1290 and 1544 since the appearances of the brick fence and road are similar to the ones of the target object. For the KCF tracker, it can locate the target object well at the beginning of the UAV tracking application. However, it also lost its target from Frame 1432 due to the similar appearance of the background, e.g., brick fence. For the TLD tracker, it is prone to lose the target object when the target object is conducting out-of-plane rotation. On the other hand, the other visual trackers can always locate the target object, especially the STRUCK and our presented tracker, which are able to track their target object within 30-pixel CLE errors. However, our presented tracker has outperformed the STRUCK tracker in the average success rate, as shown in Figure 17b, since the STRUCK tracker cannot estimate the scale of the target object.

### 4.7. Discussion

#### 4.7.1. Overall Performances

Table 3 and Table 4 show the overall performances of all visual trackers.

For the average CLE error (i.e., CLE_*Ave*_) of all image sequences, our presented tracker, the KCF and Frag trackers are ranked as No. 1, No. 2 and No. 3 in all visual trackers. For the average FPS (i.e., FPS_*Ave*_), the KCF, our presented tracker and CT tracker have achieved 149.8, 38.9 and 28.7 frames per second, resulting in rankings of No. 1, No. 2 and No. 3 among all visual trackers. For the average success rate (SR) (i.e., SR_*Ave*_) when the *ξ* is set to 0.5, our presented tracker, the KCF and Frag trackers are ranked as No.1, No. 2 and No. 3 again, especially for our presented visual tracker, which has achieved a 95.9% success rate in all image sequences.

**Figure 24:**
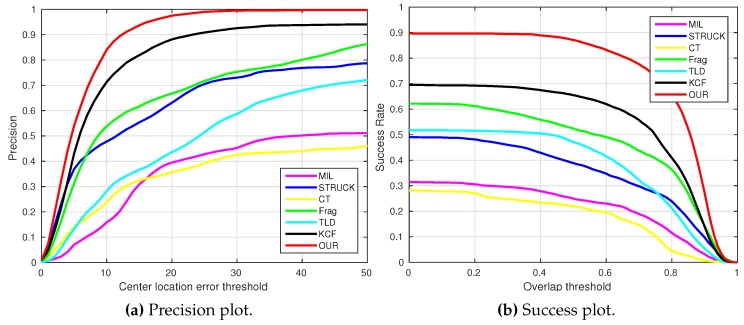
Overall performances of all visual trackers in 11,646 image frames.

Figure 24 shows the overall performances of all visual trackers in 11,646 image frames with precision and success plots. It can be seen that our presented tracker has obtained the best performance. In addition, the KCF and Frag trackers are ranked No. 2 and No. 3.

The video related to the tracking results of all visual trackers can be checked at the following YouTube link: https://youtu.be/cu9cUYqJ1P8.

#### 4.7.2. Failure Case

Our presented visual tracker cannot handle the below situations properly as it has employed the features to represent the target object: (1) strong motion blur; and (2) large out-of-plane rotation. These cases can result in cases in which the feature detector cannot detect many features, leading to imprecise estimation for the bounding box of the target object.

## 5. Conclusions

In this paper, a novel robust onboard visual tracker has been presented for long-term arbitrary 2D or 3D object tracking for a UAV. Specifically, three main modules, i.e., the GMLT, LGF and LOF modules, have been developed to obtain a reliable global-local feature-based visual model efficiently and effectively for our visual tracking algorithm, which has achieved the real-time frame rates of more than thirty-five FPS. The extensive UAV flight tests show that our presented visual tracker has outperformed the most promising state-of-the-art visual trackers in terms of robustness, efficiency and accuracy and overcome the object appearance change caused by the challenging situations. It is to be noted that the KCF tracker has achieved an average of 149.8 FPS, but its tracking precision and average success rate are less than ours in all image sequences. Additionally, the UAV can achieve good control performance with more than 20 FPS in real flights. In addition, our visual tracker does not require software or hardware stabilization systems, e.g., a gimbal platform, for stabilizing the onboard captured consecutive images throughout the UAV flights.

## Figures and Tables

**Table 1:** Challenging factors of each image sequence. MV: mechanical vibration; AF: aggressive flight; IV: illumination variation; OC: partial or full occlusion; SV: scale variation; DE: deformation, i.e., non-rigid object deformation; IR: in-plane rotation; OR: out-of-plane rotation; OV: out-of-view; CB: cluttered background. The total number of evaluated image frames in this paper is 11,646.

Sequence	Number	MV	AF	IV	OC	SV	DE	IR	OR	OV	CB
*Container*	2874	√				√			√	√	√
*Gas tank*	3869	√	√	√		√		√	√	√	√
*Moving car*	582	√						√		√	
*UGV_lp_*	1325	√			√			√		√	
*People_bw_*	934	√					√	√	√		
*People_fw_*	2062	√		√	√	√	√		√		√

**Table 2:** Main parameters in our presented visual tracker.

Parameter Name	Value	Parameter Name	Value
Bucketing configuration	10 × 8	FAST threshold	20
Sampling patch size ($S_{r}$)	48	BRIEF descriptor length ($N_{b}$)	256
Ratio threshold (*ρ*)	0.85	Local search window ($S_{h}$)	30
LGF cut-off threshold (*η*)	18	LOF cut-off threshold (*μ*)	1.5

**Table 3:** Center location error (CLE) (in pixels) and frames per second (FPS). Red, blue and green fonts indicate the best, second best and third best performances in all visual trackers. The total number of evaluated image frames in this paper is 11,646.

Sequence	MIL	STRUCK	CT	Frag	TLD	KCF	Our
*Container*	17.7	4.5	8.2	27.4	-	9.3	3.6
*Gas tank*	118.1	62.4	103.4	22.7	16.6	63.1	6.8
*Moving Car*	7.1	3.1	4.5	4.5	105.1	3.7	3.3
*UGV_lp_*	152.2	97.1	150.1	20.6	7.6	4.8	7.3
*People_bw_*	17.8	16.7	157.9	13.8	130.8	16.6	10.2
*People_fw_*	153.3	15.7	197.1	41.7	-	73.0	6.1
CLE_*Ave*_	96.4	41.6	107.1	28.4	-	18.9	6.5
FPS_*Ave*_	24.8	16.2	28.7	13.1	23.9	149.8	38.9

**Table 4:** Success rate (SR) (%) (*ξ* = 0.5). Red, blue and green fonts indicate the best, second best and third best performances in all visual trackers. The total number of evaluated image frames in this paper is 11,646.

Sequence	MIL	STRUCK	CT	Frag	TLD	KCF	Our
*Container*	62.9	62.7	62.7	62.5	81.2	96.6	99.8
*Gas tank*	25.7	61.8	24.5	90.8	85.6	62.7	97.3
*Moving car*	68.0	88.1	89.9	82.5	24.7	78.4	85.7
*UGV_lp_*	15.1	18.7	6.8	70.3	89.9	99.6	98.6
*People_bw_*	30.0	40.5	10.5	63.2	2.67	34.7	81.7
*People_fw_*	10.6	29.5	9.9	16.9	19.6	46.8	99.4
SR_*Ave*_	32.9	50.1	30.8	65.3	51.8	71.0	95.9
